# Exploratory comparisons between different anti-mitotics in clinically-used drug combination in triple negative breast cancer

**DOI:** 10.18632/oncotarget.28068

**Published:** 2021-09-14

**Authors:** Bruna Cândido Guido, Douglas Cardoso Brandão, Ana Luisa Augusto Barbosa, Monique Jacob Xavier Vianna, Lucas Faro, Luciana Machado Ramos, Fabíola Nihi, Márcio Botelho de Castro, Brenno A.D. Neto, José Raimundo Corrêa, Sônia Nair Báo

**Affiliations:** ^1^Microscopy and Microanalysis Laboratory, Department of Cell Biology, Institute of Biological Sciences, University of Brasília, Brasília 70910-900, Brazil; ^2^Laboratory of Medicinal Chemistry and Organic Syntesis, Exact and Technological Sciences Campus, State University of Goiás, Anápolis, Goiás 75001-970, Brazil; ^3^Veterinary Pathology Laboratory, Faculty of Agronomy and Veterinary Medicine, Department of Veterinary Medicine, University of Brasília, Brasília 70910-970, Brazil; ^4^Laboratory of Medicinal and Technological Chemistry, University of Brasília, Chemistry Institute, University of Brasília, Brasília 70904-900, Brazil

**Keywords:** KIF11 inhibition, Kinesin Eg5, breast cancer, adjuvant treatment, cancer progression

## Abstract

Triple-negative breast cancer (TNBC) constitutes a very aggressive type of breast cancer with few options of cytotoxic chemotherapy available for them. A chemotherapy regimen comprising of doxorubicin hydrochloride and cyclophosphamide, followed by paclitaxel, known as AC-T, is approved for usage as an adjuvant treatment for this type of breast cancer. In this study we aimed to elucidate the role of KIF11 in TNBC progression throughout its inhibition by two synthetic small molecules containing the DHPM core (dihydropyrimidin-2(1H)-ones or -thiones), with the hypothesis that these inhibitors could be an interesting option of antimitotic drug used alone or as adjuvant therapy in association with AC. For this purpose, we evaluated the efficacy of DHPMs used as monotherapy or in combination with doxorubicin and cyclophosphamide, in Balbc-nude mice bearing breast cancer induced by MDA-MB-231, having AC-T as positive control. Our data provide extensive evidence to demonstrate that KIF11 inhibitors showed pronounced antitumor activity, acting in key points of tumorigenesis and cancer progression in *in vivo* xenograft model of triple negative breast cancer, like down-regulation of KIF11 and ALDH1-A1. Moreover, they didn’t show the classic peripheral neuropathy characterized by impaired mobility, as it is common with paclitaxel use. These results suggest that the use of a MAP inhibitor in breast cancer regimen treatment could be a promising strategy to keep antitumoral activity reducing the side effects.

## INTRODUCTION

Triple-negative breast cancer (TNBC) constitutes a very aggressive subtype that accounts for approximately 12–18% of breast cancer patients [1, 2]. The stratification of patients according to their receptor status is still a cost-effective, rapid and easy way to assess the suitability of breast cancer patients to targeted treatments [3]. Since TNBCs patients do not express estrogen receptor (ER), progesterone receptor (PR) and human epidermal growth factor receptor (HER-2), they are not eligible for hormone or HER2 target therapies, and so, few chemotherapy options remain available for them [4, 5].

Chemotherapeutic agents currently approved for these breast tumors that are unsuitable for targeted therapies, typically target DNA synthesis and repair, therefore tending to have more side effects. In general, drug combinations commonly work better for TNBC patients than monotherapy because different drugs can achieve the heterogeneous cell mass throughout different pathways [6]. This treatment is usually given as a combination of drugs that associates DNA intercalators, that inhibit DNA and RNA synthesis, such as doxorubicin [7]; alkylating agents that irreversibly crosslink DNA leading to apoptosis, such as cyclophosphamide; and mitotic inhibitors, such as taxanes [3].

A chemotherapy regimen comprising of doxorubicin hydrochloride (Adriamycin) and cyclophosphamide, followed by paclitaxel (Taxol), known as AC-T, is approved for usage as an adjuvant treatment for breast cancer. Besides the great proportion of patients that relapse and evolve resistance, leading to poor overall survival, there are still severe issues related to side effects of these drugs resulting in a huge clinical need for the identification of effective targets in TNBC [8].

Paclitaxel is an antineoplastic agent indicated as first-line and subsequent therapy for the treatment of advanced carcinoma of ovarian, breast, lung and also for Kaposi’s sarcoma [9, 10]. Its antineoplastic effect is due to its activity of hyper-stabilization of microtubules structures and by its direct interaction to β tubulin as well [11]. The resulting complex abolishes cell’s ability to use its cytoskeleton in a flexible way, inducing mitotic arrest and leading to cell death in a subset of the arrested population [12]. However, since this dynamic instability of microtubules of shortening and lengthening is essential for important cell functions besides cell division, like cargo transportations, many side effects come along with the administration of this drug [13]. Some of the most common adverse effects are low blood counts, both red and white cells, and platelets as well, what can increase the risk of infection, anemia and bleeding, arthralgias and myalgias, nausea, vomiting and severe peripheral neuropathy.

Microtubule-associated proteins (MAPs) have emerged as an alternative and promising anticancer target since their inhibition would block mitosis without producing neurotoxicity [14]. Some of these MAPs, like Kinesin Spindle Protein (KSP), also known as KIF11 or Kinesin Eg5, are not expressed in terminally differentiated cells, such as neurons; therefore, toxicities such as peripheral neuropathy are not predictable with KIF11 inhibitors. This class of proteins plays a specific and essential role in the assembly of the bipolar spindle and chromosome segregation through ATP hydrolysis. KIF11 inhibition leads to monoastral microtubule formation, which culminates in cell cycle arrest at mitosis and ultimately in cell death [15, 16]. Besides its essential roles, it was recently shown that KIF11 overexpression might contribute to tumorigenesis [17] and is associated with a poorer prognosis in a broad range of cancers, such as breast cancer [18], hepatocellular carcinoma [19], laryngeal squamous cell carcinoma [20], astrocytic neoplasm [21] and renal cell carcinoma [22] among others, being suggested as a potential prognostic biomarker. Additionally, overexpression of KIF11 in transgenic mice induced the development of several types of malignancies [23]. Considering the already mentioned correlation between high levels of KIF11 and worse prognostic in different cancer types, several inhibitors for this motor protein are currently being studied in clinical trials [17]. However, despite the great results obtained in preclinical studies using KIF11 inhibitors there are still some problems being faced in clinics, like the occurrence of neutropenia being the dose-limiting factor and the emergence of resistance. Thus, vast majority of inhibitors that went to clinical trials have failed to show efficacy mainly when used as monotherapy suggesting that new treatment strategies could be used to succed [17, 24, 25].

In this study we aimed to elucidate the role of KIF11 in TNBC progression throughout its inhibition by two DHPM (dihydropyrimidin-2(1H)-ones or -thiones) based synthetic small molecules, previously characterized by our research group [26, 27]. For this purpose, we evaluated the efficacy of DHPMs used as monotherapy and in combination with other drugs approved for breast cancer treatment, in balbc-nude mice bearing breast cancer induced by MDA-MB-231 cells inoculation. Treatment with DHPMs was able to impair the correct chromosome segregation during metaphase in tumor cells, leading them to death by apoptosis. We have also shown that there is a correlation between KIF11 expression levels and inhibition of tumor progression. We demonstrated that this class of DHPMs worked better when used as adjuvant therapy, presenting similar results of growth tumor inhibition when compared to those induced by paclitaxel treatment, besides to down-regulate KIF11 and ALDH1-A1 expression, effects not observed in paclitaxel treatment group. This effectiveness was achieved without the classic peripheral neuropathy presented by taxanes suggesting that the use of KIF11inhibitors as adjuvant therapy in invasive breast cancer treatment could be a promising strategy.

## RESULTS

### KIF11 inhibitors didn’t show any toxicity related to weight loss, motor capacity or systemic toxicity

Animals of all experimental groups were monitored with regards to their weight, behavior and clinical aspects. Animals body weight were assessed prior the beginning of treatment and twice a week throughout the experiment period as an important indicator of toxicity.

**Figure 1:**
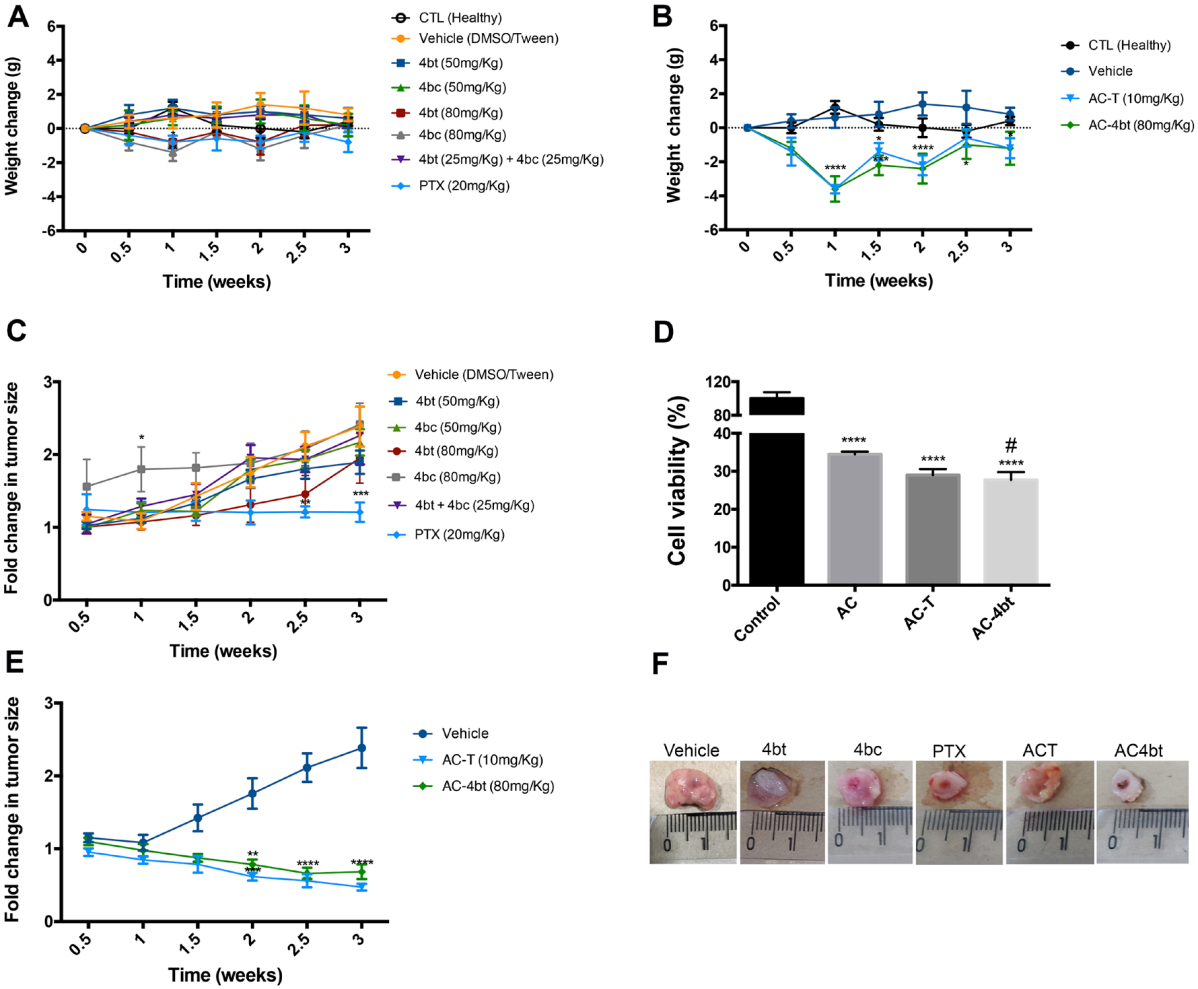
Body weight and tumor volume monitoring during monotherapy and drug combination therapy. (**A**) Body weight of animals submitted to monotherapy, administered with drug diluent (vehicle group), 4bt (50 or 80 mg/Kg), 4bc (50 or 80 mg/Kg), 4bt + 4bc (25 mg/Kg each) or paclitaxel (20 mg/Kg) or (**B**) to drug combination, doxorubicin (10 mg/Kg) + cyclophosphamide (100 mg/Kg) in a single dose, followed by three doses of KIF11 inhibitor 4bt (80 mg/Kg) or paclitaxel (10 mg/Kg) was monitored twice a week. Tumor volume for monotherapy (**C**) and drug combination (**D**) was also checked during all treatment time. (**E**) An *in vitro* survival assay with MDA-MB-231 cells was performed to evaluate if the association of paclitaxel or 4bt could potentiate antitumor effect of drug combination (doxorubicin and cyclophosphamide) treatment. (**F**) A representative image of tumor for each experimental group. Lines and columns represent mean ± SEM of five animals used in each experimental group. ^*^ *p* < 0.05, ^**^ *p* < 0.01, ^***^ *p* < 0.001, ^****^ *p* < 0.0001 as compared to control group; ^#^ *p* < 0.05 compared to AC treatment group.

When considering monotherapy treatment, it was observed a weight reduction upon treatment with 4bt and 4bc (80 mg/Kg) and PTX (20 mg/Kg) at time point of 1 week after the beginning of treatment, however, after this time no significant weight change in all experimental groups was observed compared to the control healthy group (Figure 1A).

When drug combination was administered in animals, a pronounced decrease in weight was observed one week after application of doxorubicin associated to cyclophosphamide (AC) and, after a time, animals recovered (Figure 1B). Additionally, animals treated with AC showed loss of appetite and considerable reduction in their level of activity, and some animals died due to the high toxicity of the treatment. The animals that recovered were randomized into two experimental groups to follow up the therapeutic regimen with cycles of paclitaxel or KIF11 inhibitor (4bt), but the administration of either of these two drugs did not cause any further reduction in the animals’ weight (Figure 1B).

Although paclitaxel used as adjuvant therapy did not cause an expressive weight loss in animals since its dose was lower than the used for monotherapy, 10 mg/Kg and 20 mg/Kg respectively, significant behavioral changes were observed in both groups (Supplementary Videos 1 and 2). A few minutes after administration, animals showed reduced motor capacity, and dragged their hind legs to move around. Even with the administration of higher doses of 4bt compared to paclitaxel dose, 80 mg/Kg *versus* 10 or 20 mg/Kg, respectively, no similar adverse effect was observed neither in the treatment with 4bt as monotherapy nor when it was used with drug combination (Supplementary Videos 3 and 4).

Additionally, a complete blood count (CBC) and biochemical profile to evaluate drug toxicity to critical organs, such as liver and kidneys, were performed. As already described on literature, paclitaxel caused a decrease in blood counts, red and white cells and platelets as well. Our results corroborate these already mentioned findings. Treatment with paclitaxel (20 mg/Kg) caused the most prominent reduction in hematocrit values, as can be seen in Table 1. Paclitaxel group also presented a reduced red cell count, although not statistically significant and this alteration was responsible for the decrease observed in hemoglobin values for this treatment group (Table 1). As expected, paclitaxel also caused a reduction in total white blood cells (WBC) compared to healthy control animals (2139.39 ± 684.75 and 4447.26 ± 1434.71, respectively) (Supplementary Table 1). White blood cells also presented alterations upon treatment in some other experimental groups compared to healthy animals. We observed a considerable increase in absolute lymphocytes number in vehicle and 4bt group, and a pronounced decrease in paclitaxel treated animals (Supplementary Table 1). The red cell distribution width (RDW) presented statistically significant increase upon treatment with paclitaxel and drug combinations AC-T and AC-4bt.

**Table 1:** Red blood cell analysis of Balb-c nude mice bearing breast tumor

	Red cells (10^6^/mm^3^)	Hemoglobin (g/dL)	Hematocrit (%)	MCV (fl)	MCH (pg)	MCHC (g/dL)	RDW (%)
CTL Healthy	9.48 ± 0.65	14.50 ± 0.81	49.26 ± 2.73	52.52 ± 0.69	15.46 ± 0.22	29.44 ± 0.32	19.15 ± 0.67
Vehicle	9.90 ± 0.31	14.60 ± 0.23	51.09 ± 1.62^**^	51.58 ± 0.43	14.74 ± 0.31	28.57 ± 0.60	20.06 ± 0.32
4bt (50 mg/Kg)	9.38 ± 0.23	14.23 ± 0.4	48.53 ± 1.62	51.75 ± 0.88	15.18 ± 0.23	29.32 ± 0.45	19.68 ± 0.40
4bc (50 mg/Kg)	9.29 ± 0.3	14.07 ± 0.51	47.43 ± 2.06^**^	51.07 ± 081^*^	15.13 ± 0.4	29.67 ± 0.68	19.65 ± 0.56
4bt (80 mg/Kg)	8.97 ± 0.34	13.56 ± 0.56	45.13 ± 1.83^****^	50.30 ± 0.16^***^	15.13 ± 0.21	30.03 ± 0.37	19.70 ± 0.22
4bc (80 mg/Kg)	9.75 ± 0.4	14.57 ± 0.56	48.77 ± 2.11	50.04 ± 0.68^****^	14.94 ± 0.12	29.88 ± 0.47	20.34 ± 0.41
4bt+4bc (25 mg/kg)	9.57 ± 0.62	14.48 ± 0.84	49.23 ± 2.63	51.48 ± 0.71	15.14 ± 0.29	29.39 ± 0.54	20.00 ± 0.89
Paclitaxel 20 mg/kg	8.15 ± 0.08	12.80 ± 0.14^*^	42.56 ± 0.62^****^	52.23 ± 0.42	15.73 ± 0.05	30.07 ± 0.12	22.85 ± 0.98^****^
AC-T	8.72 ± 0.15	13.42 ± 0.28	44.57 ± 0.99^****^	51.10 ± 0.71	15.40 ± 0.18	30.10 ± 0.46	21.22 ± 0.13^**^
AC-4bt	9.23 ± 0.49	14.06 ± 0.80	46.94 ± 2.35^***^	50.84 ± 0.71^*^	15.24 ± 0.10	29.98 ± 0.36	21.46 ± 0.36^***^

MCV: Mean corpuscular volume; MCH: Mean corpuscular hemoglobin; MCHC: mean corpuscular hemoglobin concentration; RDW: red cell distribution width. Data represent mean ± SEM. ^*^ *p* < 0.05, ^**^ *p* < 0.01, ^***^ *p* < 0.001, ^****^ *p* < 0.0001 as compared to control group (CTL Healthy).

With regards to biochemical parameters, only Lactic Acid Dehydrogenase (LDH) showed significant changes among treated groups compared to control of healthy animals (Table 2). Paclitaxel and both drug combinations were the ones that caused more pronounced decrease in LDH levels compared to control (Table 2).

**Table 2:** Biochemical analysis of Balb-c nude mice bearing breast tumor

	Creatinine (mg/dL)	LDH (U/L)	ALP (U/L)	GGT (U/L)	AST (UI/L)	ALT (UI/L)	Urea (mg/dL)
CTL Healthy	< 0,2	673.20 ± 154.01	109.40 ± 8.24	1	115.00 ± 16.70	30.00 ± 2.45	68.80 ± 7.47
Vehicle	< 0,2	527.00 ± 118.82^***^	79.00 ± 12.44	1.20 ± 0.40	98.40 ± 26.31	25.00 ± 1.10	56.60 ± 4.88
4bt (50 mg/Kg)	< 0,2	677.60 ± 84.90	63.40 ± 2.42	1.00 ± 0.63	152.00 ± 37.35	28.60 ± 3.67	60.00 ± 4.15
4bc (50 mg/Kg)	< 0,2	712.75 ± 107.63	67.75 ± 8.47	1.00 ± 0.71	132.50 ± 26.69	27.50 ± 5.89	53.50 ± 4.92
4bt (80 mg/Kg)	< 0,2	552.20 ± 133.08 ^*^	75.80 ± 10.93	< 1	150.00 ± 40.31	34.20 ±2.64	68.40 ± 4.76
4bc (80 mg/Kg)	< 0,2	537.80 ± 122.98^**^	93.80 ± 14.69	1	118.80 ± 29.38	29.40 ± 4.50	63.60 ± 3.14
4bt+4bc (25 mg/kg)	< 0,2	705.80 ± 331.33	73.80 ± 10.89	1.60 ± 2.25	218.00 ± 139.16*	41.60 ± 20.95	53.00 ± 4.38
Paclitaxel (20 mg/kg)	< 0,2	464.50 ± 50.5^****^	86.50 ± 10.5	< 1	98.50 ± 21.50	21.50 ± 1.50	59.00 ± 2.00
AC-T	< 0,2	424.00 ± 70.21^****^	77.00 ± 6.60	< 1	108.75 ± 19.18	21.50 ± 1.80	54.25 ± 1.92
AC-4bt	< 0,2	398.20 ± 98.17^****^	93.40 ± 9.99	< 1	100.80 ± 19.96	28.20 ± 4.12	60.60 ± 7.17

LDH: Lactate dehydrogenase; ALP: alkaline phosphatase; GGT: Gamma-glutamyl transferase; AST: aspartate aminotransferase; ALT: alanine transaminase. Data represent mean ± SEM. ^*^ *p* < 0.05, ^**^ *p* < 0.01, ^***^ *p* < 0.001, ^****^ *p* < 0.0001 as compared to control group (CTL Healthy).

### 4bt used as monotherapy holds tumor growth

To evaluate antitumor efficacy of KIF11 inhibitors used as monotherapy, nude mice bearing breast tumor were administered intraperitoneally (i.p.) with different doses of 4bt and 4bc (50 mg/kg or 80 mg/kg) or paclitaxel (20 mg/Kg), used as positive control for comparison. Tumor growth was monitored during all the treatment time. Figure 1C shows that 4bt presented a significant antiproliferative activity and at the highest concentration tested (80 mg/kg) it was able to inhibit tumor growth mainly until 2.5 weeks from the beginning of treatment when compared to vehicle control. Animals treated with 4bc inhibitor didn’t show any considerable difference in tumor volume compared to untreated animals.

A combination of the two inhibitors (25 mg/Kg of each) was also tested to evaluate any possible synergic antitumor effect, but no significant changes were observed (Figure 1C).

### 4bt inhibitor used as adjuvant therapy potentiates the suppressor effect of drug combination in breast tumor *in vitro* and *in vivo*

Based on the results obtained in the monotherapy experiments, where 4bt presented an interesting antitumor and antiproliferative activity, we aimed to evaluate the efficacy of 4bt used as an adjuvant therapy in combination with doxorubicin and cyclophosphamide (AC), a treatment regimen choice used in association with paclitaxel for invasive triple negative breast cancer. Our proposal was to verify the possibility of using KIF11 inhibitor in this treatment regimen in replacement for paclitaxel.

In order to address this goal, we performed an *in vitro* survival assay to assess if the association of paclitaxel or 4bt would potentiate antitumor effect of treatment in MDA-MB-231 cells. Cells treated with AC-only for 24 h showed 34.2 % ± 0.27 of viability (Figure 1D). With the addition of paclitaxel or 4bt, the viability decreased to 29.70 % ± 0.66 and 27.70 % ± 0.84, respectively. Based on these results we performed *in vivo* tests to evaluate if these drugs combination would have the same effectiveness in the presence of the tumor microenvironment.

*In vivo* experiments showed that 4bt had similar activity to paclitaxel when used in combination with doxorubicin and cyclophosphamide significantly decreasing the tumor volume (Figure 1E and 1F). Tumor sizes were considerably reduced mainly after 1.5 weeks of the beginning of treatment, when the adjuvant therapy was introduced.

As can be noticed, 4bt activity was comparable to the one obtained with paclitaxel treatment, however animals from the first treatment group did not show any characteristic signal of peripheral neuropathy or impaired mobility, commonly presented by microtubule targeting drugs, as can be seen in Supplementary Videos 1 and 3.

Treatment with KIF11 inhibitor decreases intratumoral necrotic area To assess antiproliferative and antitumor effects of the treatments proposed on this work, histopathologic analysis of tumor sections of all animal groups were performed after HE staining.

**Figure 2:**
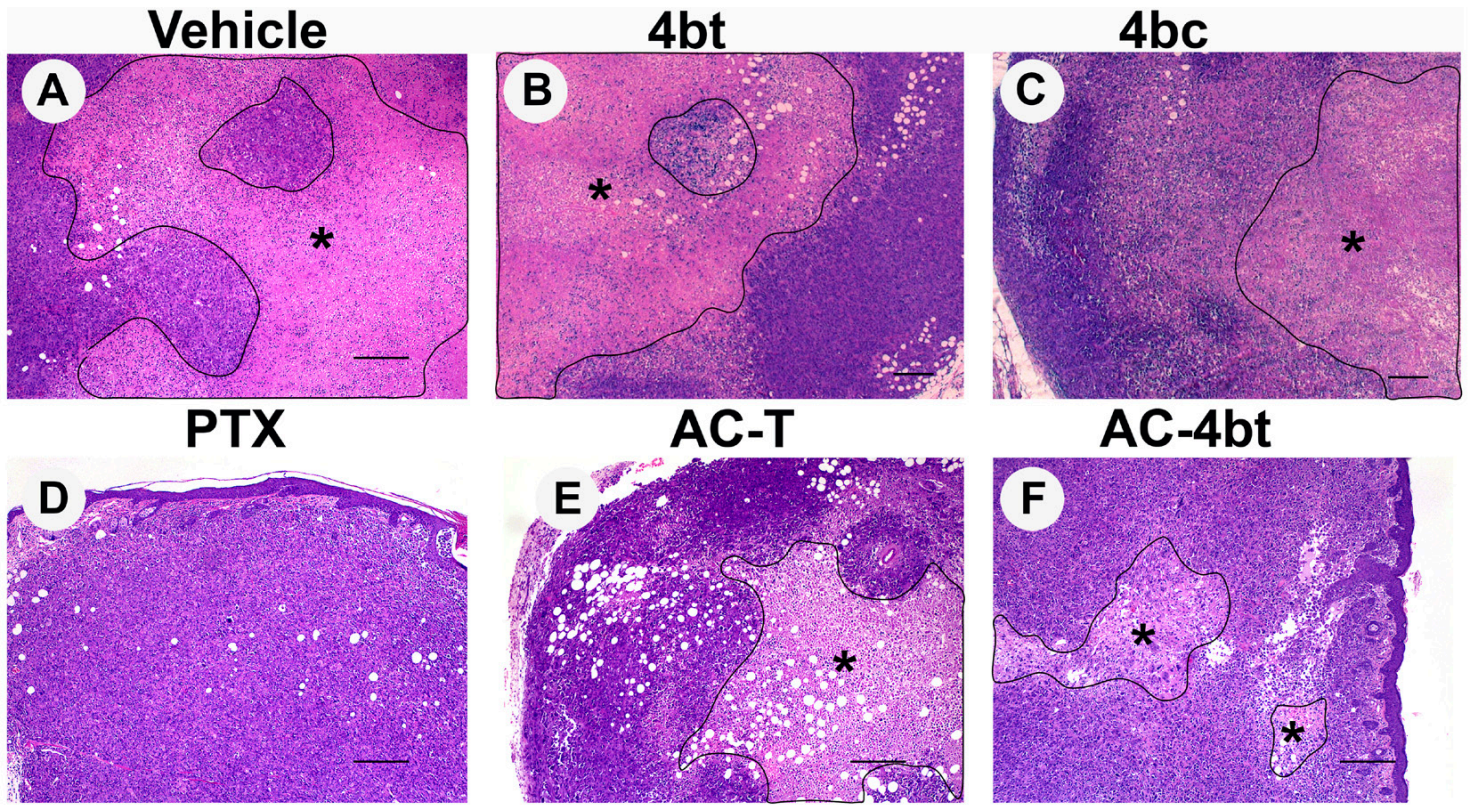
Histopathological analysis of tumor sections upon monotherapy or drug combination treatments. Representative images of paraffin-embedded sections of tumors stained with H&E from the different experimental groups administered intraperitoneally (i.p.) with: (**A**) Vehicle, (**B**) 4bt (80 mg/Kg), (**C**) 4bc (80 mg/Kg), (**D**) paclitaxel (20 mg/Kg), (**E**) Doxorubicin (10 mg/Kg) + Cyclophosphamide (100 mg/Kg) followed by paclitaxel (10 mg/Kg) (AC-T) and (**F**) Doxorubicin (10 mg/Kg) + Cyclophosphamide (100 mg/Kg) followed by KIF11 inhibitor 4bt (AC-4bt). Treatment with 4bt used as monotherapy or in drug combination prevent exacerbated tumor growth leading to a decrease in the extension of the central necrotic area (outlined and marked with asterisk). Bars: 250 μm (A, D, E and F) and 200 μm (B and C).

As can be seen on Figure 2A, vehicle group presented a large intratumoral necrotic area showing a pattern of fast growing of the tumor cells during the period of study. When monotherapy with 4bt, 4bc or paclitaxel was performed (Figure 2B–2D), we observed a reduction in the size of necrotic area, and for 4bt treatment (80 mg/kg), this reduction was associated with a slight decrease in tumor volume, assessed by caliper, during treatment time (Figure 1C and 1F). Association of KIF11 inhibitor 4bt in drug combination treatment regimen besides to cause a decrease in tumor volume it also caused a considerable reduction in intratumoral necrotic area (Figure 2F), even better than AC-T drug combination (Figure 2E), showing important antiproliferative activity in this tumor model.

To assess the tumor burden in one of the most common sites of metastasis for breast cancer, paraffin-embedded sections of lungs were analyzed in a blinded manner, however none metastasis or any other histopathological changes were observed in nude-mice bearing breast tumor of all experimental groups compared to the control healthy group as shown in Supplementary Figure 1. This indicates that the established time for tumor growth before the beginning of treatment could be not enough for tumor metastasis and also that none of the treatment caused any toxicity for lungs.

### 4bt decreases intratumoral KIF11 expression and leads to monoastral spindle formation *in vivo*

KIF11 has been shown to be overexpressed in a broad range of cancer what contributes to tumorigenesis, being associated with a worse prognostic [18]. These findings lead us to question if KIF11 inhibitors could somehow regulate its expression besides to impair its activity. To test this hypothesis, we performed a western blot analysis of MDA-MB-231 cells control and treated with KIF11 inhibitors 4bt and 4bc at two different concentrations. Interestingly, it was observed that 4bt and 4bc regulated the expression levels of this protein, and this regulation was dose-dependent (Supplementary Figure 2). Thus, we aimed to evaluate the intratumoral expression of KIF11 in the *in vivo* model used in this study.

**Figure 3:**
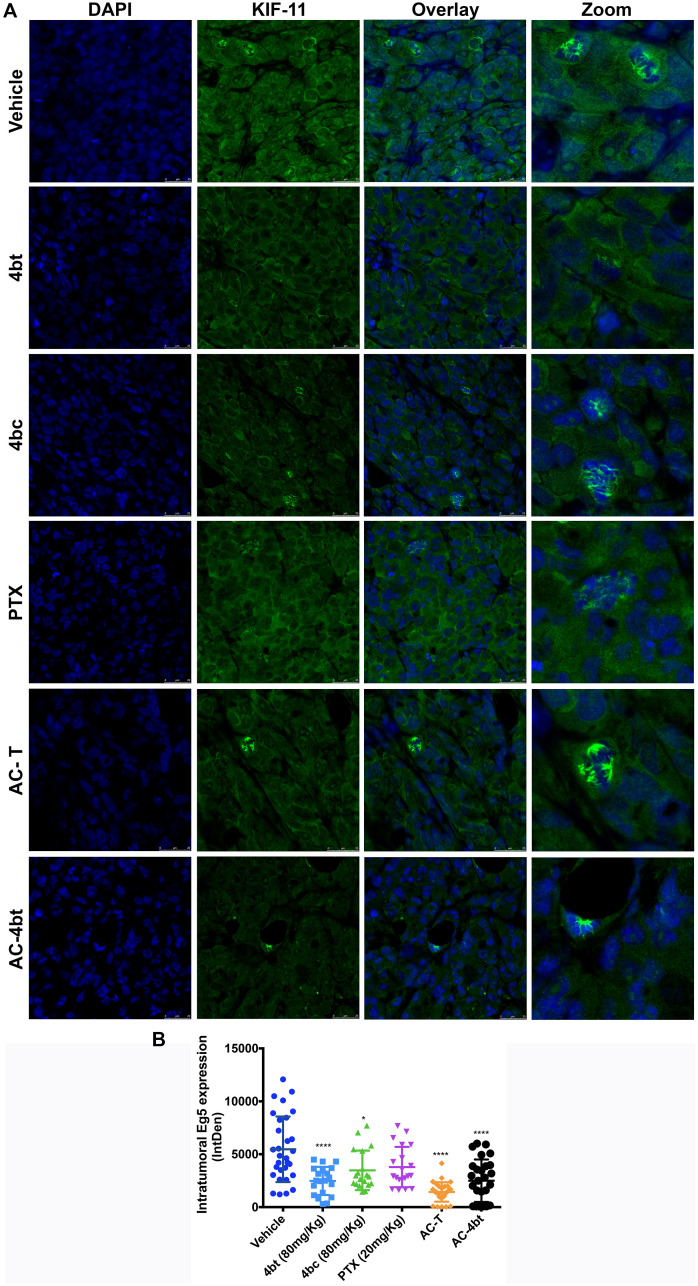
Analysis of intratumoral KIF11 expression. (**A**) Tumor sections from the different experimental groups administered intraperitoneally (i.p.) with: Vehicle, 4bt (80 mg/Kg), 4bc (80 mg/Kg), paclitaxel (20 mg/Kg), Doxorubicin (10 mg/Kg) + Cyclophosphamide (100 mg/Kg) followed by paclitaxel (10 mg/Kg) (AC-T) and Doxorubicin (10 mg/Kg) + Cyclophosphamide (100 mg/Kg) followed by KIF11 inhibitor 4bt (AC-4bt) were immunostained for KIF11 (green) and genetic material (blue). (**B**) Integrated Density calculated by using sets containing 10 random images of each experimental group. 4bt or drug combinations were able to significantly decrease intratumoral KIF11 expression levels. Data represent mean ± SEM of three independent experiments. ^*^ *p* < 0.05, ^****^ *p* < 0.0001 as compared to control group (vehicle). Bars: 25 μm.

To examine the effects of the inhibitors on intratumoral KIF11 expression, tumor sections of treated and vehicle animals were immunostained for KIF11 analysis. As can be seen in Figure 3A and 3B, tumors of vehicle animals showed superexpression of KIF11. This expression can be observed both in nucleus, even in cells that are not dividing, and in cytoplasm. It is also possible to visualize a great number of dividing cells presenting bipolar and multipolar spindle, as expected for MDA-MB-231 cells [9, 28].

Monotherapy with KIF11 inhibitors led to a less intense expression of intratumoral KIF11, mainly with administration of 4bt, in which we can see KIF11 expression occurring predominantly in cytoplasm and a smaller number of cells dividing. Paclitaxel alone didn’t cause any change on KIF11 levels. Drug combination of both AC-T and AC-4bt showed a relevant role in downregulating KIF11 expression levels, and it is also possible to note that association of 4bt induced monoastral spindle formation as it was already described for *in vitro* treatment [26] (Figure 3A and 3B).

### Treatment with KIF11 inhibitor induces apoptotic cell death and decreases ALDH1-A1 expression

To evaluate the mechanism behind tumor growth inhibition observed by tumor measure analysis caused by DHPMs, we assessed proliferation index and apoptotic cell death by Ki67 staining of tumor sections and TUNEL assay, respectively.

**Figure 4:**
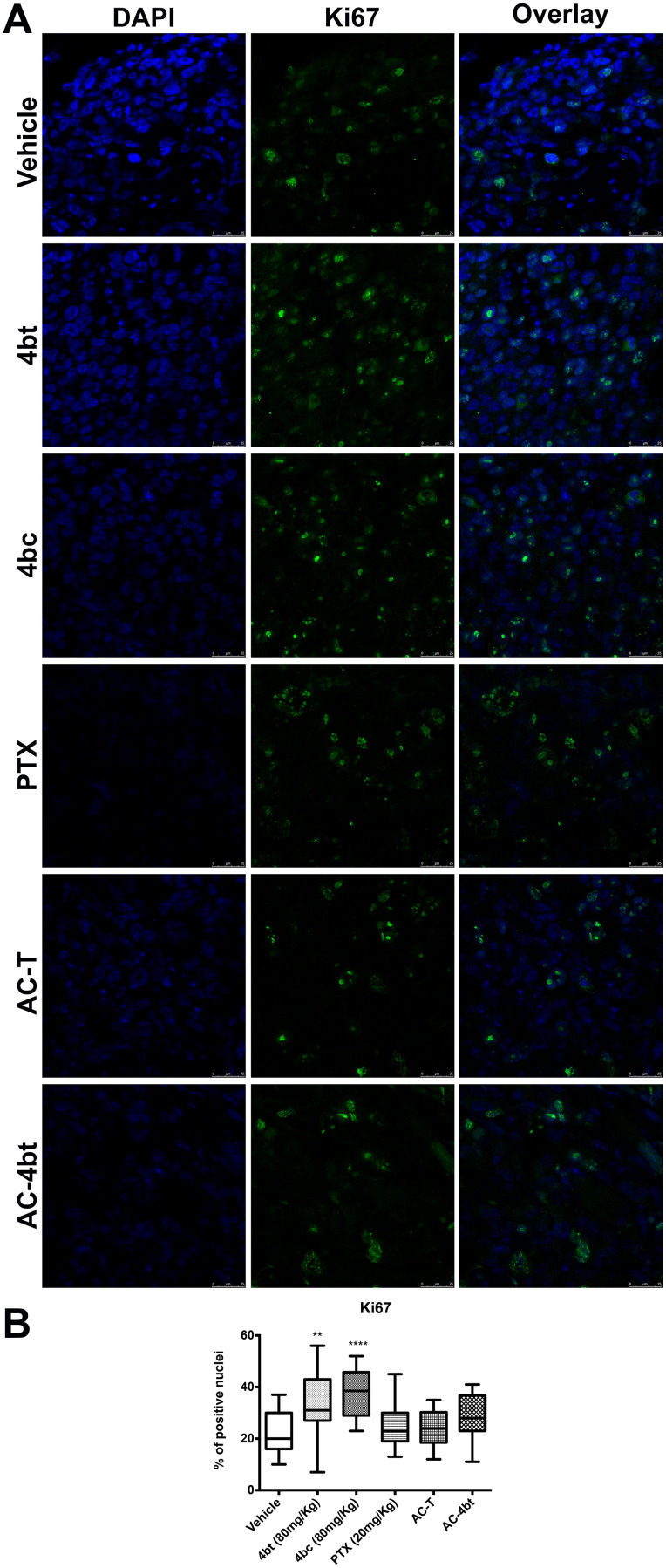
Proliferation analysis of tumor sections. (**A**) Tumor sections from the different experimental groups administered intraperitoneally (i.p.) with: Vehicle, 4bt (80 mg/Kg), 4bc (80 mg/Kg), paclitaxel (20 mg/Kg), Doxorubicin (10 mg/Kg) + Cyclophosphamide (100 mg/Kg) followed by paclitaxel (10 mg/Kg) (AC-T) and Doxorubicin (10 mg/Kg) + Cyclophosphamide (100 mg/Kg) followed by KIF11 inhibitor 4bt (AC-4bt) were immunostained for Ki67 (green) and genetic material (blue). (**B**) Percentage of Ki67 positive nuclei for each experimental group. Data represent mean ± SEM of three independent experiments. ^**^ *p* < 0.01, ^****^ *p* < 0.0001 as compared to control group (vehicle). Bars: 25 μm.

**Figure 5:**
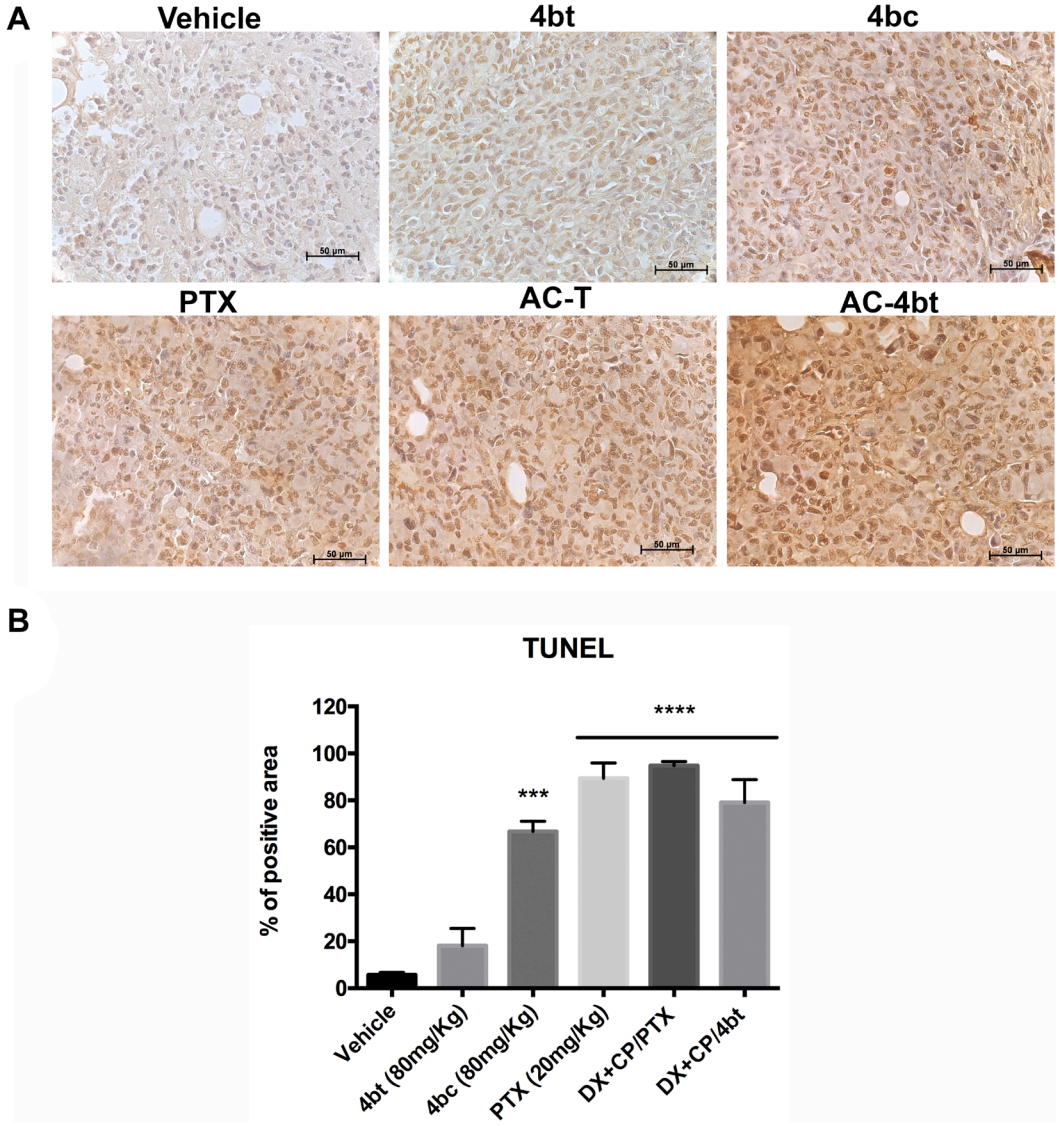
TUNEL analysis for apoptosis detection in tumor sections of xenografted Balb-c/nude mice. (**A**) Representative images of TUNEL assay of tumor sections from the different experimental groups administered intraperitoneally (i.p.) with: Vehicle, 4bt (80 mg/Kg), 4bc (80 mg/Kg), paclitaxel (20 mg/Kg), Doxorubicin (10 mg/Kg) + Cyclophosphamide (100 mg/Kg) followed by paclitaxel (10 mg/Kg) (AC-T) and Doxorubicin (10 mg/Kg) + Cyclophosphamide (100 mg/Kg) followed by KIF11 inhibitor 4bt (AC-4bt). (**B**) Quantitative analyses of apoptotic cell area for each experimental group. Data represent mean ± SEM of three independent experiments. ^***^ *p* < 0.001, ^****^ *p* < 0.0001 as compared to control group (vehicle). Bars: 50 μm.

Monotherapy with KIF11 inhibitors 4bt and 4bc were responsible for a slight increase in Ki67 expression by breast tumor cells compared to vehicle group, as can be seen on Figure 4A and 4B. Drug combination using AC associated to 4bt as adjuvant therapy didn’t cause significant changes in proliferation rate being similar to the results obtained by AC-T, as shown in Figure 4A and 4B. However, based on tumor volume monitored during treatment time we could assume that mainly drug combination was effective in decrease tumor growth. These data were corroborated by TUNEL analysis, where we can see that positive control paclitaxel and both drug combinations, AC-T and AC-4bt, substantially increase positive area staining showing a considerable apoptotic activity caused by these treatments (Figure 5A and 5B).

In a previous work of our research group, we evaluated the role of several KIF11 inhibitors on cancer stem cell (CSC) population in MDA-MB-231 cells throughout the analysis of CD44 and CD24 expression. We found that specifically these two inhibitors selected for this work, 4bt and 4bc, were able to considerably reduce CD44^+^/CD24^-^ population. ALDH1-A1, an isozyme associated to cancer stem-cells in many solid tumors, is another breast CSC marker, involved in self-renewal, differentiation and self-protection. High ALDH1 expression is associated with early relapses; metastasis development and therapy resistance being correlated with poor clinical outcomes in breast cancer patients [29–35]. Moreover, it was shown a positive correlation between KIF11 and ALDH1 expression in MCF-7 and SKBR-3 cells [36]. So, we aimed to verify if KIF11 inhibitors could also reduce ALDH1-A1 contributing to a better response to treatment.

**Figure 6:**
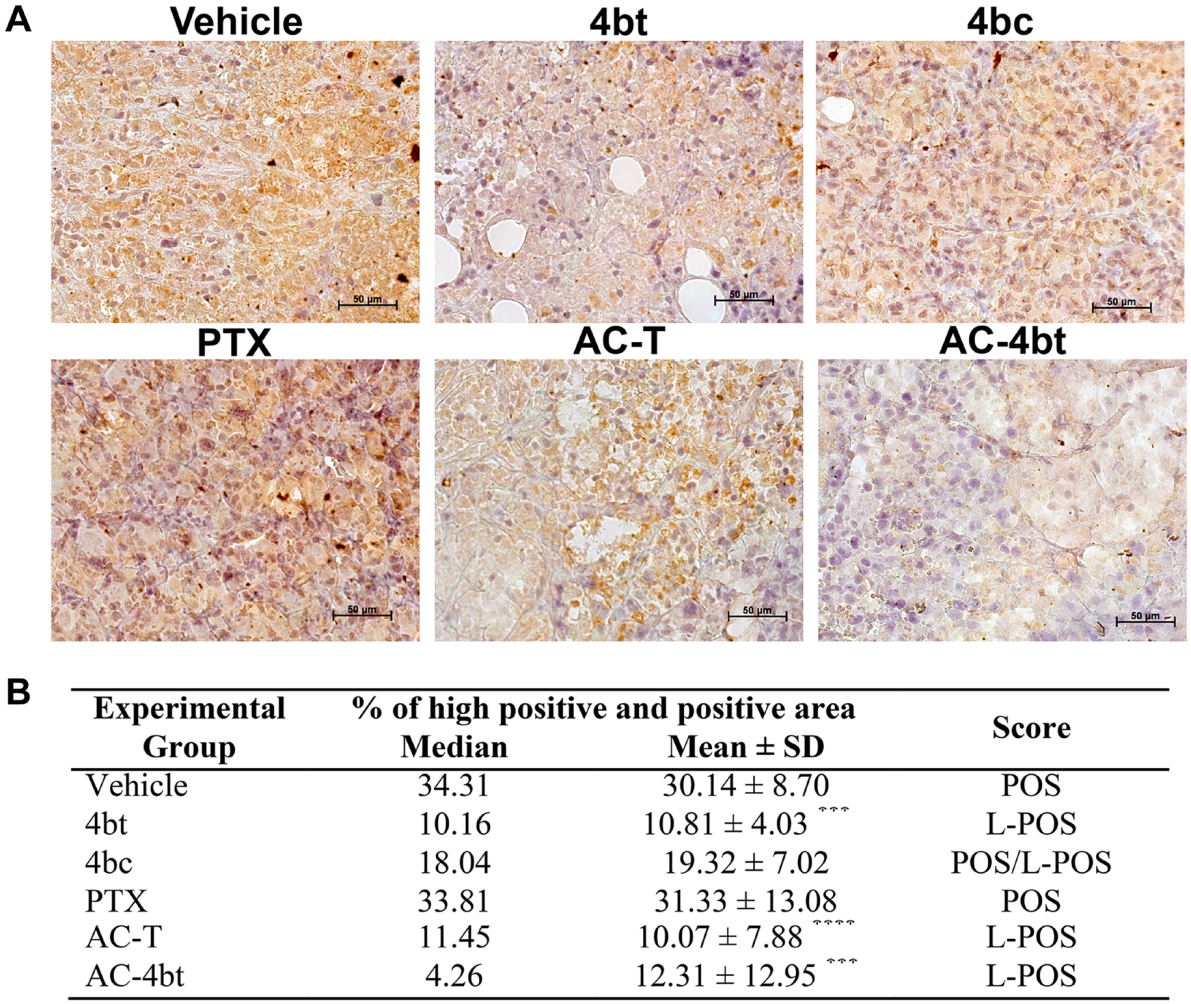
ALDH1-A1 expression analysis in tumor sections of xenografted Balb-c/nude mice. (**A**) Representative images of ALDH1-A1 immunostaining of tumor sections from the different experimental groups administered intraperitoneally (i.p.) with: Vehicle, 4bt (80 mg/Kg), 4bc (80 mg/Kg), paclitaxel (20 mg/Kg), Doxorubicin (10 mg/Kg) + Cyclophosphamide (100 mg/Kg) followed by paclitaxel (10 mg/Kg) (AC-T) and Doxorubicin (10 mg/Kg) + Cyclophosphamide (100 mg/Kg) followed by KIF11 inhibitor 4bt (AC-4bt). (**B**) Quantitative analyses highly positive and positive area with indication of the score for each experimental group. L-POS: low positive; POS: positive. Data represent mean ± SEM of three independent experiments. ^***^ *p* < 0.001, ^****^ *p* < 0.0001 as compared to control group (vehicle). Bars: 50 μm.

In ALDH1-A1 analysis we could note that 4bt was able to down regulate its expression in an expressive and significant way both when used as monotherapy and when used as adjuvant therapy with drug combination (Figure 6A) leading the median of the percentage of high positive and positive area from 34.31, in vehicle group, to 10.16 (4bt) and 4.26 (AC-4bt), and wide part of tumor was scored as low-positive. Treatment with paclitaxel and 4bc didn’t cause significant changes in ALDH1-A1 expression being similar to vehicle group with a positive score (Figure 6B).

## DISCUSSION

TNBC patients normally receive as standard care neoadjuvant chemotherapy with a combination of mitotic inhibitor, as taxanes, and a DNA intercalator, as anthracyclines. Despite the effectiveness of this therapeutic regimen, about 30 to 50% of patients still relapse with emergence of drug resistant clones, leading to poor overall survival [1, 6].

Taxanes function by binding to tubulin, an essential protein for cell division, motility, cell shape and intracellular transport. The disruption of microtubule dynamics caused by these drugs are responsible for activating the spindle checkpoint leading to cell death. However, since microtubules have other important roles in cell biology, these agents can also cause peripheral neuropathy as an adverse event by interfering with microtubule-based axonal transport [37, 38].

In this context KIFs have emerged as a prominent e promising strategy to fight tumor cells. Several recent studies have reported that KIF11 is highly expressed in several types of tumors and is often associated with chemotherapeutic drug resistance and poor prognosis [19, 21, 22, 39]. KIF11, or Kinesin Eg5, is the most well-studied protein of this family in clinical setting. Its overexpression generates genome instability and carcinogenesis in mouse models and it is also responsible for tumor angiogenesis [23, 40].

Despite the great results on preclinical studies, some disappointing results in clinical trials with KIF11 inhibitors were observed over the past years. There are some main reasons for this, like the fact that the doubling time of human tumors is much longer than xenografts, suggesting a small proportion of mitotic cells in human tumors compared to animal xenografts [17]. Another reason is the development of drug resistance due to the emergence of point mutations in enzymatic domain of Eg5 that constitutes part of the drug-binding site [41]. Besides, as already mentioned, we have the neutropenia as the main dose-limiting toxicity [24, 42]. Even with the unsuccessful results in some clinical tests, the results in preclinical tests added to the absence of neurotoxic effects, indicate that this class of inhibitors can still be a good therapeutic option, especially if used in combination with other drugs.

In this work we propose the substitution of the taxane (paclitaxel) in the AC-T therapeutic breast cancer regimen with a KIF11 inhibitor, in the hope of preserving antitumoral activity while reducing toxicity.

Our data showed that 4bt, one of the KIF11 inhibitors evaluated on this work, was able to hold tumor growth when used as monotherapy, however it presented more pronounced antitumor effects when used in combination with other drugs, as already pointed for other KIF11 inhibitors [43, 44]. The great advantage in using this class of inhibitor is to minimize the intense and unpleasant adverse events causes by some class of drugs like taxanes. Neutropenia is the most common adverse effect presented by KIF11 inhibitors already analyzed on clinical trials [45, 46]. Ispinesib was the first and is the most well characterized KIF11 inhibitor to enter in clinical trials [47]. In phase I trial in breast cancer, Ispinesib showed relevant antitumor activity and stabilization of the disease [48]. Among the reported side effects were neutropenia, increase in hepatic transaminases and diarrhea [46]. Besides, ispinesib was well-tolerated and had no indication of neurotoxicity [49, 50].

In our study, we verified some level of neutropenia in animals treated with 4bt (80 mg/Kg), however this alteration was not statistically significant, as it is common in clinical trials with this class of inhibitors [24], what makes it an attractive therapeutic option. Also, we could verify some evident neurotoxicity effects induced by paclitaxel a few minutes after administration as observed on supplemental videos. To achieve a considerable breast tumor growth inhibition on our model, a concentration of 20 mg/Kg of paclitaxel was intraperitoneally administered. Animals that received this treatment lost their motor control and could not move their hind legs normally. None similar effect was verified in DHPMs treatment groups, even at the highest concentrations tested (80 mg/Kg). The low expression of KIF11 in non-tumoral tissues that are not proliferating results in a lower toxicity of KIF11-targeted therapy when compared to traditional anti-mitotic therapies [51].

Several works have shown a correlation between high expression levels of Eg5 and poor prognosis in breast cancer [18], hepatocellular carcinoma [19], thymic malignancies [52], laryngeral squamous cell carcinoma [20], renal cell carcinoma [22] and in non-muscle invasive bladder urothelial carcionoma [39]. So, we aimed to evaluate if DHPMs could affect the expression levels of intratumoral KIF11 besides to interfere in its activity as elucidated in a previous work of our research group [26]. 4bt both alone and in combination with other drugs, was able to decrease intratumoral KIF11 expression *in vivo* upon intraperitoneally administration. These data differ from the results obtained *in vitro*, since in the latter, 4bt inhibitor caused a slight increase in the expression of KIF11 when the IC_50_ concentration was used, and in the *in vivo* assay, both 4bc and 4bt decreased the expression of this protein. This difference may be due to both the drug concentration that reaches the tumor and the influence of the tumor microenvironment in the *in vivo* model.

The effect of this inhibitor was kept even with the influence of tumor microenvironment as we can observe the formation of monoastral spindle in dividing cells.

As KIF11 is involved with breast cancer development, drug resistance [53] and poor prognosis, the control of its expression can improve results obtained with chemotherapy, being responsible for better outcomes. In addition to this, 4bt also exerts growth inhibitory effect *in vivo*, being responsible for induction of apoptosis cell death in tumor and leading to a slower growth of tumor cells that presented smaller areas of necrosis in the core of the tumor.

With regards to Ki-67 expression, DHPMs cause a cell cycle arrest at G2/M as previously reported by our group [26]. Treatment of MDA-MB-231 cells *in vitro* with 4bt and 4bc inhibitors didn’t lead to a cell cycle arrest at G2/M because of the marked cytotoxic effect and rapid elimination of cells. However, our *in vivo* experiments pointed for a slower induction of apoptosis, confirmed by the great amount of intratumoral cells presenting monoastral spindle, so we have the occurrence of cells arrested in G2/M phase. It is known that Ki-67 levels are higher in G2 phase and mitosis [54, 55], so this result in the increase in Ki-67 levels in tumors of xenografted mice treated with 4bt and 4bc alone, could be reflecting this phase of arrest, and not an absence of antiproliferative effect, since tumors showed some volume reduction, mainly with 4bt treatment.

Despite having the best antitumor activity *in vitro*, 4bc inhibitor was not able to reduce the tumor volume in xenografted animals. This may have occurred due to the concentration used in the *in vivo* tests, which had its maximum dose limited by drug solubility. As shown in previously published work of our research group, this inhibitor presented a considerably higher IC_50_, which indicates that higher doses may be also necessary for it to maintain its activity *in vivo*. These data also justify the fact that 4bc treatment has shown interesting results regarding Ki67 expression and TUNEL analysis,

Another interesting finding was the down-regulation of ALDH1-A1 expression upon treatment with 4bt both as monotherapy and in combination with AC. This corroborates with our *in vitro* data in which 4bt considerably decrease cancer stem cells (CSC) represented by CD44^+^/CD24^-^ population [26]. ALDH1-A1 is an important marker of CSCs and its high expression is correlated with poorer overall survival in breast cancer patients [31, 56]. Pei and collaborators (2019) showed that self-renewal of breast cancer cells is enhanced by endogenous KIF11 through activating Wnt/β-catenin signaling pathway contributing to the breast cancer stem cell features. They have shown that reduced levels of CSC markers as Oct4, Nanog, ALDH1 and CD44 were observed in breast cancer cells with silenced KIF11 [36]. Moreover, KIF11 expression was positively correlated to ALDH1 in oesophageal squamous cell carcinoma [57].

It was also reported that ALDH1-A1 and ALDH2-A1 have a role in the conversion of activated cyclophosphamide (4-hydroperoxycyclophosphamide) to the inactive excretory carboxyphosphamide providing to CSC drug protection and radiation resistance [58, 59]. This interesting result was not observed in animals treated with paclitaxel.

In conclusion, our study provides extensive evidence to demonstrate that KIF11 inhibitor 4bt showed pronounced antitumor activity, acting in key points of tumorigenesis and cancer progression in *in vivo* xenograft model of triple negative breast cancer. These considerations imply that KIF11 inhibitors may represent a promising strategy to be used as adjuvant therapy in breast cancer treatment regimen and may improve TNBC patients’ outcomes showing considerably fewer side effects. Furthermore, this inhibitor has shown other interesting roles for tumor inhibition, like down-regulation of KIF11 and ALDH1-A1, particularly when combined with existing treatments.

## MATERIALS AND METHODS

### Animals

Animal work was performed using 8-week-old female Balb-c/nude mice. Animals were kept in the Bioassay Laboratory at Catholic University of Brasília housed in five animals per cage under 12 h light–dark cycles at a controlled temperature (23°C ± 2°C), with water and food *ad libitum*. After tumor inoculation, mice were randomized into treatment groups (*n* = 5 per group). This study was approved by the Committee on Animal Research and Ethics of the Catholic University of Brasília (CEUA/UCB – Protocol 009/16).

### Tumor induction and treatment

Mice were anesthetized with ketamine (80 mg/kg of body weight) and xylazine (20 mg/kg of body weight) solution. 2 × 10^6^ MDA-MB-231 cells in PBS/20% type I Collagen (100 μL) were inoculated in the fourth right mammary fat pad. After tumor establishment, about 5 weeks after inoculation, mice were randomized into experimental groups (*n* = 5 per group), and treatment started.

Experiments were performed in two phases, in the first one, DHPMs were used as monotherapy, and experimental groups comprised to: 1) Control (Healthy animals); 2) Vehicle (1% Tween 80/6.25% DMSO in PBS, pH 7.4); 3) 4bt (50 mg/kg); 4) 4bc (50 mg/Kg); 5) 4bt (80 mg/kg); 6) 4bc (80 mg/Kg); 7) 4bt + 4bt (25 mg/Kg each); 8) paclitaxel (20 mg/Kg - diluted in PBS from a solution of 6 mg/mL in purified polyoxyl castor oil and 49.7% (v/v) of dehydrated alcohol and sodium metabisulfite). Doses and schedules were determined based on previous works with the same class of compounds [38, 60] and maximal dose of 80 mg/Kg was established taking in to account the solubility of the inhibitors used in this work.

**Figure 7:**
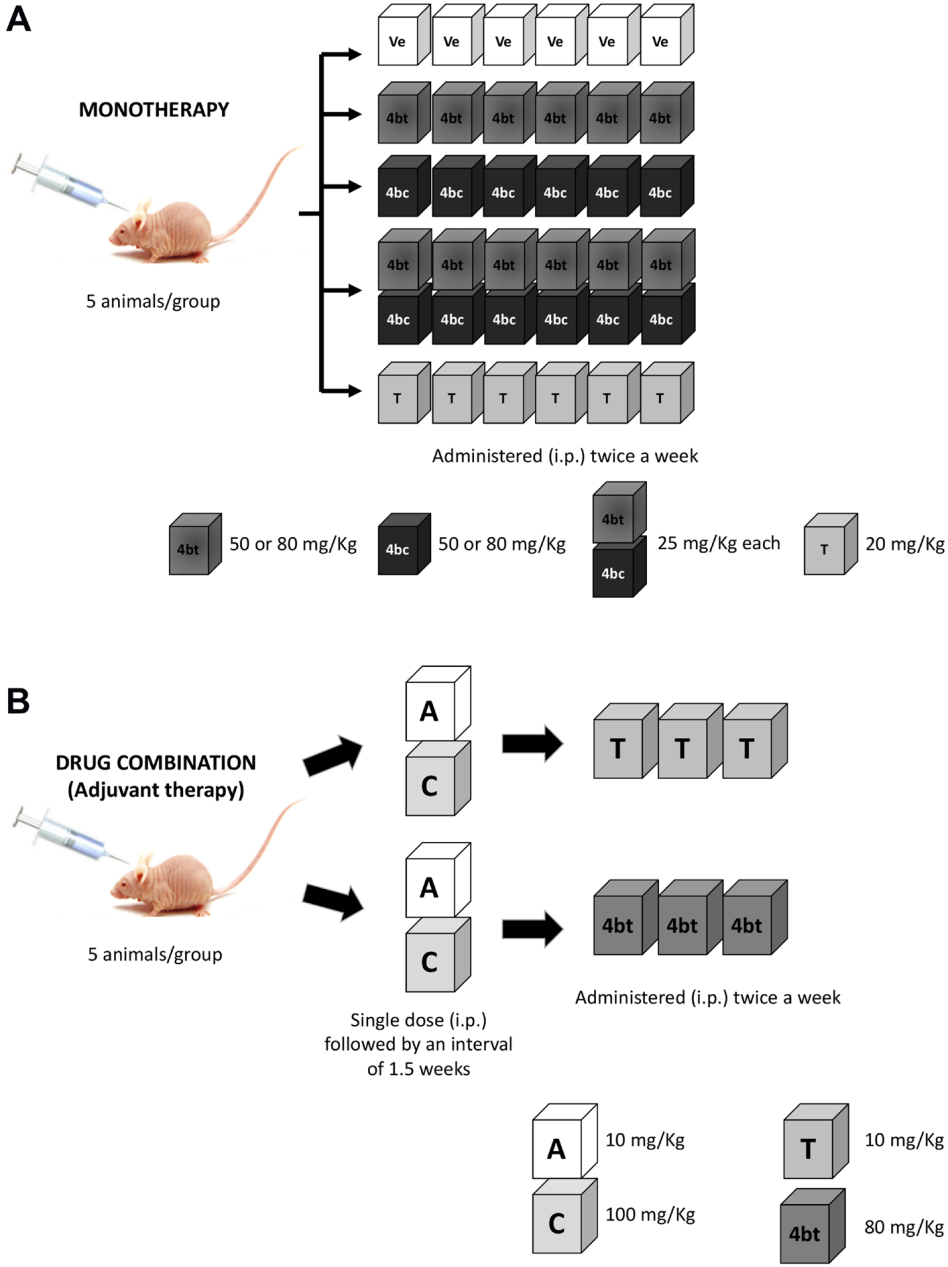
Scheme of therapeutic regimen applied in Balb-c/nude mice bearing breast cancer. (**A**) For monotherapy, animals were administered i.p. with six doses of 4bt, 4bc (50 or 80 mg/Kg), 4bt + 4bc (25 mg/Kg each) or paclitaxel (20 mg/Kg) twice a week. (**B**) For drug combination, animals received one dose of doxorubicin (10 mg/Kg) + cyclophosphamide (100 mg/Kg) i.p. and after 1.5 weeks three doses of 4bt (80 mg/Kg) or paclitaxel (10 mg/Kg) were administered twice a week.

In the second phase, the DHPM that showed effectiveness in *in vivo* treatment, 4bt, was used as adjuvant therapy in combination with standard treatment regimen for invasive breast cancer: 1) AT-C: Doxorubicin (Adriamycin) (DX - 10 mg/Kg) + Cyclophosphamide (CP – 100 mg/Kg) in a single dose, followed by three doses of paclitaxel (10 mg/Kg twice a week), used as positive control; or 2) AT-4bt: Doxorubicin (Adriamycin) (DX - 10 mg/Kg) + Cyclophosphamide (CP – 100 mg/Kg) in a single dose, followed by three doses of 4bt (80 mg/Kg twice a week). All treatments were administered intraperitoneally (i.p) (Figure 7).

### Weight and tumor growth monitoring

Tumor length (l) and width (w) were measured with a caliper and recorded every 3–4 days and tumor volume was calculated using the following formula V (mm^3^) = (l × w^2^)/2. Animal weight was also monitored with help of a digital scale twice a week.

### Complete Blood Count (CBC) and biochemical exams

Blood samples were collected in appropriate tubes for hematological (WBC, NEUT, LYPMPH, MONO, EOS, BASO, RBC, HGB, HCT, MCV, MCH, MCHC, RDW, PLT MPV) and biochemical (Creatinine, LDH, ALP, GGT, ALT, AST and urea) parameters analysis. All the tests were performed in collaboration by Sabin Diagnostic Laboratory.

### Histological analysis

Tumors and lungs were fixed in 10% buffered formalin and processed for paraffin embedding. Processed material was sectioned at 5 μm, and sections were stained with hematoxylin & eosin (HE) for analysis under the light microscope Axiovert (Zeiss, Germany). Tumor and lung sections were analyzed for general histopathological assessment.

### Analysis tumor cell apoptosis by TUNEL assay

The level of apoptosis in tumor sections was determined by TUNEL assay. TUNEL staining was performed on paraffin-embedded sections using Click-iT™ TUNEL Colorimetric IHC Detection Kit (Invitrogen, Carlsbad – USA) according to the manufacturer’s protocol. Briefly, the sections were dewaxed, rehydrated, and immersed in 4% paraformaldehyde fixative solution for 15 minutes. They were then washed and incubated with proteinase K for 20 min at room temperature. The slides were washed with PBS for 5 minutes, and immersed in 4% paraformaldehyde fixative solution again for 5 minutes at room temperature. Slides were then washed with PBS (twice, for 5 minutes) and rinsed with deionized water. Samples were incubated in TdT Reaction Buffer for 10 minutes at 37°C. The TdT reaction buffer was gently removed using paper towels and slides were incubated with TdT Reaction Mixture for another hour at 37°C in a humidified chamber. After being rinsed with PBS, slides were immersed in 2X SSC for 15 minutes, in order to fully quench TdT reaction. Slides were washed with 1X Click-iT™ TUNEL Colorimetric Wash solution and incubated with the Click-iT™ TUNEL Colorimetric Reaction cocktail for 30 minutes at 37°C, protected from light. Slides were then rinsed respectively with PBS, 1X Click-iT™ TUNEL Colorimetric Wash solution and deionized water. Samples were then incubated with 1X Streptavidin-Peroxidase Conjugate at room temperature for 30 minutes in a humidified chamber and later washed 3 times with PBS before being rinsed with deionized water. Finally, slides were developed using the manufacturer’s DAB Reaction Mixture (1:20 dilution of DAB Chromogen in DAB Substrate Buffer), washed with deionized water and counterstained with hematoxylin for analysis with light microscope Axiovert (Zeiss, Germany).

After acquisition, images were submitted to a semi-quantitative analysis using the IHC Profiler plug-in (National Institute of Health) from ImageJ software. Images were submitted to a deconvolution process, and histogram profiles, corresponding to pixel intensity as well as a stain-positivity percentage value derived from a scoring system based on optical density, were automatically generated. Areas rated as highly positive or positive were considered for analysis.

### Analysis of intratumoral KIF 11, ALDH1-A1 and Ki67 expression

In order to evaluate the role of KIF-11 inhibitors in some key features for tumor progression and development, we analyzed tumor sections for the levels of KIF-11, ALDH1-A1 and Ki67 expression by immunofluorescence (IF) or immunohistochemistry. Immunostaining was performed on 5 μm sections of formalin-fixed paraffin-embedded (FFPE) tissue. Tissue slides were baked, deparaffinized in xylene and passed through graded alcohols for rehydration. Antigen retrieval was achieved submitting samples to a 10 mM Trisodium Citrate solution, pH 6.0 in a steam pressure cooker at 100°C for 10 minutes. Slides were then left immersed in the Trisodium Citrate solution at room temperature for 20 minutes before being washed with PBS/0.025% Triton. The area around each section was delimited by a hydrophobic liquid blocker (PAP-Pen) in order to assure homogeneous distribution of reagents and antibodies. Slides were pretreated with 1% skimmed milk/2,5% BSA/8%Fetal Bovine Serum+PBS for 30 minutes, washed and subsequently incubated with the respective primary antibodies (rabbit anti-KIF11 – 1:50; rabbit anti-ALDH1A1 – 1:100 - and rabbit anti Ki-67 – 1:50 - polyclonal antibodies) overnight at 4°C, inside a humidified chamber.

Slides were then washed in PBS/0.025% Triton, incubated at room temperature in the dark with (1:500) Alexa Fluor 488-Anti-rabbit secondary antibody, for KIF-11 and Ki67, or (1:250) Goat anti-rabbit HRP, for ALDH1-A1 for 1 hour, washed three times with PBS. Slides destined for immunofluorescence were stained with DAPI (300 nM) and mounted with Fluoromount-G mounting medium (Electron Microscopy Sciences, Hatfield – USA) and specimens observed under a laser scanning confocal microscope TCS SP5 (Leica, Wetzlar, HE, Germany). Slides for ALDH1-A1 analysis were counterstained with hematoxylin and analyzed under light microscope Axiovert (Zeiss, Germany).

For ALDH1-A1 quantification of positive areas, IHC Profiler plug-in was used as previously described for TUNEL analysis.

### Viability assay

As we hypothesized that KIF11 inhibitors could potentiate antitumor effect when used in combination with conventional drugs for invasive breast cancer, we performed an *in vitro* viability assay using MDA-MB-231 cells to test this hypothesis. 5 × 10^3^ MDA-MB-231 cells were plated in 96-well plates and treated with DHPM (4bt), the KIF11 with best results in the first phase of the *in vivo* tests, or paclitaxel in combination with doxorubicin and cyclophosphamide. Cells were treated first with doxorubicin + cyclophosphamide (0.12 μM and 2.5 μM, respectively) for 24 h. After this period of incubation, fresh medium was added to the wells for 48 h followed by addition of IC_50_ of paclitaxel (0.15 μM) or 4bt (17.91 μM) for 72 h. Cytotoxicity was determined using PrestoBlue Cell Viability Reagent according to the manufacturer’s instructions. Absorbance readings were measured by a spectrophotometer. Cell viability was normalized to control (vehicle only).

### Statistical analysis

Data were expressed as mean ± SEM. Statistical analyses and significance were determined by ANOVA with post-hoc comparison by Bonferroni test using GraphPad PRISM software version 6.0. A *P* value of <0.05 was considered statistically significant.

## SUPPLEMENTARY MATERIALS













## Abbreviations

AC-T: Drug combination of Doxorubicin Hydrochloride (Adriamycin) (A), Cyclophosphamide (C) and Paclitaxel (Taxol) (T)
ALDH1-A1: Aldehyde dehydrogenase 1
TNBC: Triple Negative Breast Cancer
KIF11: Kinesin Family Member 11
DHPM: dihydropyrimidin-2(1H)-ones or -thiones
MAP: Microtubule Associated Proteins
PTX: Paclitaxel
TUNEL: Terminal deoxynucleotidyl transferase dUTP nick end labeling
CBC: Complete Blood Count
LDH: Lactate dehydrogenase
ALP: alkaline phosphatase
GGT: Gamma-glutamyl transferase
ALT: alanine transaminase
AST: aspartate aminotransferase
MPV: mean platelet volume
MCV: mean corpuscular volume
MHC: Mean corpuscular hemoglobin
MCHC: mean corpuscular hemoglobin concentration
RDW: red cell distribution width
WBC: white blood cells
