# Correction: Exploratory comparisons between different anti-mitotics in clinically-used drug combination in triple negative breast cancer

**DOI:** 10.18632/oncotarget.28266

**Published:** 2022-09-28

**Authors:** Bruna Cândido Guido, Douglas Cardoso Brandão, Ana Luisa Augusto Barbosa, Monique Jacob Xavier Vianna, Lucas Faro, Luciana Machado Ramos, Fabíola Nihi, Márcio Botelho de Castro, Brenno A.D. Neto, José Raimundo Corrêa, Sônia Nair Báo

**Affiliations:** ^1^Microscopy and Microanalysis Laboratory, Department of Cell Biology, Institute of Biological Sciences, University of Brasília, Brasília 70910-900, Brazil; ^2^Laboratory of Medicinal Chemistry and Organic Syntesis, Exact and Technological Sciences Campus, State University of Goiás, Anápolis, Goiás 75001-970, Brazil; ^3^Veterinary Pathology Laboratory, Faculty of Agronomy and Veterinary Medicine, Department of Veterinary Medicine, University of Brasília, Brasília 70910-970, Brazil; ^4^Laboratory of Medicinal and Technological Chemistry, University of Brasília, Chemistry Institute, University of Brasília, Brasília 70904-900, Brazil

**This article has been corrected:** In Supplementary Figure 1, there is an accidental image overlap of panel C across panel D. The corrected figure with the new panel D, obtained from the original data, is shown below. The authors declare that these corrections do not change the results or conclusions of this paper.

Original article: Oncotarget. 2021; 12:1920–1936. 1920-1936. https://doi.org/10.18632/oncotarget.28068

**Supplementary Figure 1:**
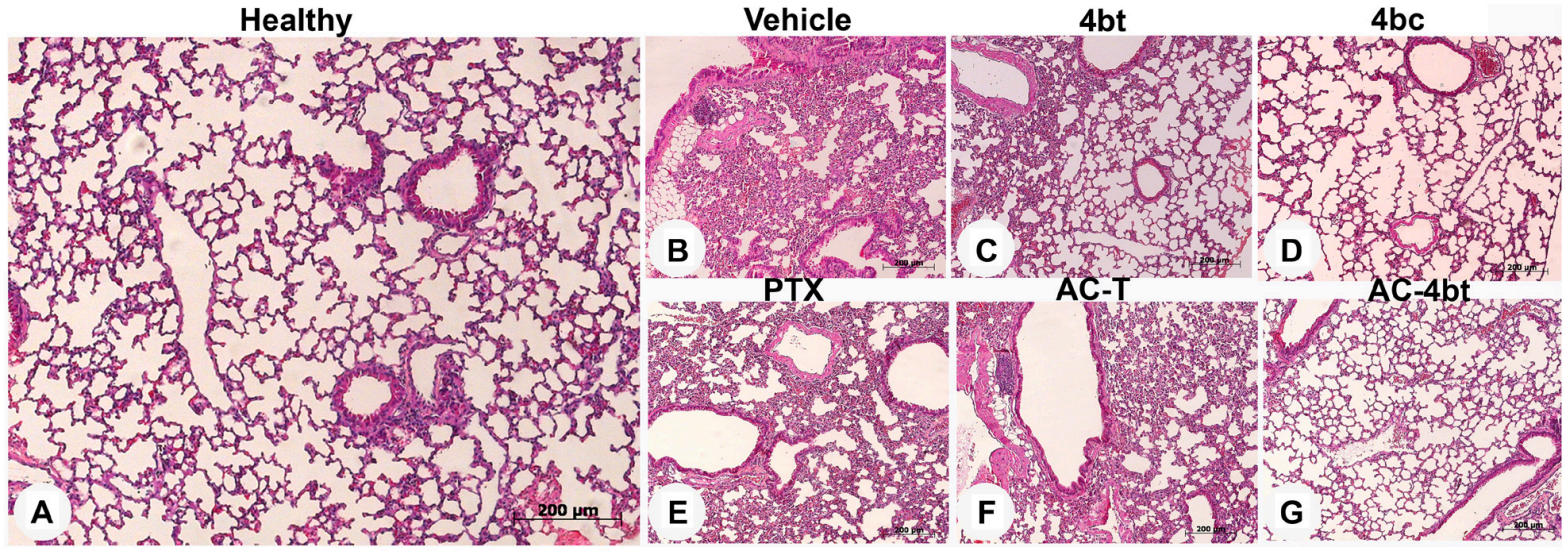
Histopathological analysis of lung sections in control animal and upon monotherapy or drug combination treatments. Representative images of paraffin-embedded sections of lungs stained with H&E from the different experimental groups: (**A**) Healthy control, or administered intraperitoneally (i.p.) with: (**B**) vehicle, (**C**) 4bt (80 mg/Kg), (**D**) 4bc (80 mg/Kg), (**E**) paclitaxel (20 mg/Kg), (**F**) Doxorubicin (10 mg/Kg) + Cyclophosphamide (100 mg/Kg) followed by paclitaxel (10 mg/Kg) (AC-T) and (**G**) Doxorubicin (10 mg/Kg) + Cyclophosphamide (100 mg/Kg) followed by KIF11 inhibitor 4bt (AC-4bt). Metastatic foci or morphological alterations were not observed in any experimental group compared to control of healthy animals. Bars: 200 μm.

