# Supplementary material for: Exploratory comparisons between different anti-mitotics in clinically-used drug combination in triple negative breast cancer
Source: Oncotarget. 2021 Sep 14;12(19):1920–36. doi: 10.18632/oncotarget.28068 (PMC8448514; doi:10.18632/oncotarget.28068)
Supplement: Supplementary file 1 [file oncotarget-12-1920-s001.pdf]

Exploratory comparisons between different anti-mitotics in clinically-used drug combination in triple negative breast cancer

SUPPLEMENTARY MATERIALS

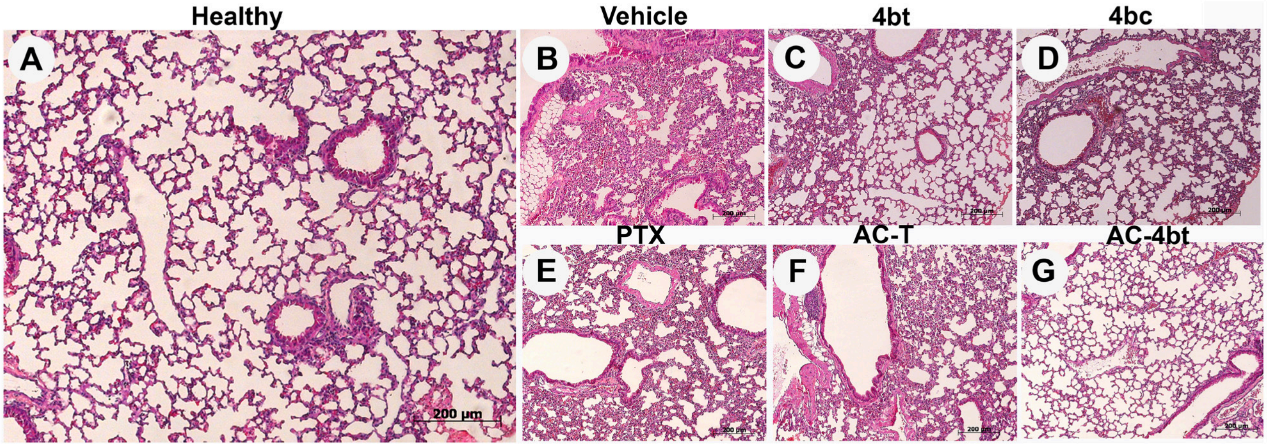

Supplementary Figure 1: Histopathological analysis of lung sections in control animal and upon monotherapy or drug combination treatments. Representative images of paraffin-embedded sections of lungs stained with H&E from the different experimental groups: (**A**) Healthy control, or administered intraperitoneally (i.p.) with: (**B**) vehicle, (**C**) 4bt (80 mg/Kg), (**D**) 4bc (80 mg/Kg), (**E**) paclitaxel (20 mg/Kg), (**F**) Doxorubicin (10 mg/Kg) + Cyclophosphamide (100 mg/Kg) followed by paclitaxel (10 mg/Kg) (AC-T) and (**G**) Doxorubicin (10 mg/Kg) + Cyclophosphamide (100 mg/Kg) followed by KIF11 inhibitor 4bt (AC-4bt). Metastatic foci or morphological alterations were not observed in any experimental group compared to control of healthy animals. Bars: 200 µm.

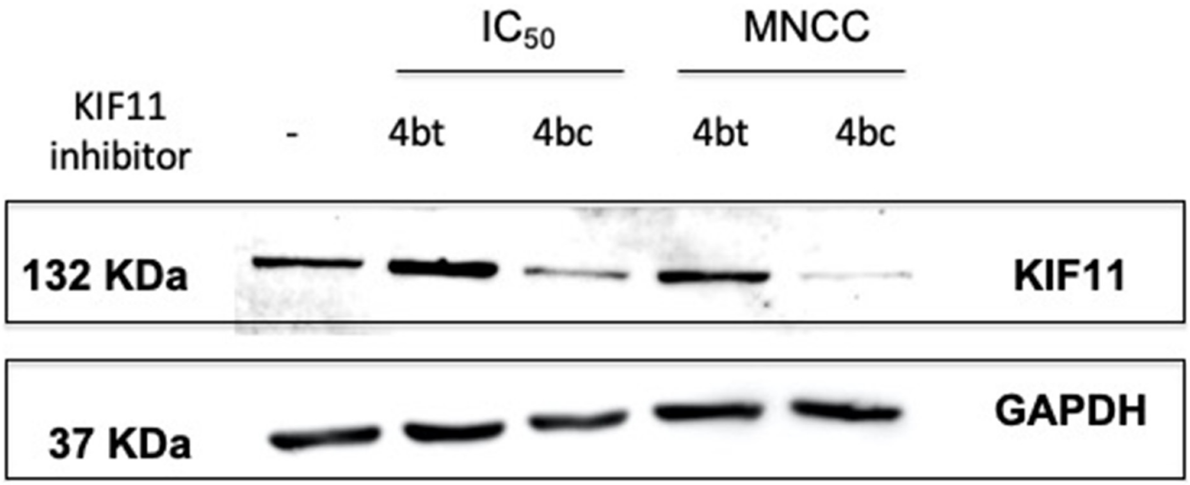

Supplementary Figure 2: ***In vitro*** **KIF11 expression evaluation upon KIF11 inhibitors treatment.** Western blot analysis of endogenous KIF11 expression in MDA-MB-231 cells control or treated for 24 h with KIF11 inhibitors 4bt and 4bc at IC_{50} (17.91 µM and 249.5 µM, respectively) or Maximum Non-Cytotoxic Concentration (MNCC) (0.8 mM and 1.0 mM, respectively).

Supplementary Video 1: Behavioral observation of animals treated with paclitaxel. See Supplementary Video 1

Supplementary Video 2: Behavioral observation of animals treated with drug combination of doxorubicin + cyclophosphamide followed by paclitaxel (AC-T). See Supplementary Video 2

Supplementary Video 3: Behavioral observation of animals treated with KIF11 inhibitor 4bt. See Supplementary Video 3

Supplementary Video 4: Behavioral observation of animals treated with drug combination of doxorubicin + cyclophosphamide followed by 4bt (AC-4bt). See Supplementary Video 4

Supplementary Table 1: With blood cell analysis of Balb-c nude mice bearing breast tumor. See Supplementary Table 1
