# Supplementary material for: Exploratory comparisons between different anti-mitotics in clinically-used drug combination in triple negative breast cancer
Source: Oncotarget. 2021 Sep 14;12(19):1920–36. doi: 10.18632/oncotarget.28068 (PMC8448514; doi:10.18632/oncotarget.28068)
Supplement: Supplementary file 6 [file oncotarget-12-1920-s006.docx]

**Supplementary Table 1: With blood cell analysis of Balb-c nude mice bearing breast tumor**

|  | **WBC** | **Lymphocytes (%)** | **Neutrophils (%)** | **Eosinophils (%)** | **Basophils (%)** | **Monocytes (%)** | **Lymphocytes  (Abs)** | **Neutrophils  (Abs)** | **Eosinophils  (Abs)** | **Basophils  (Abs)** | **Monocytes  (Abs)** | **Platelets × 10^3^/mm^3^** | **MPV  (fl)** |
| --- | --- | --- | --- | --- | --- | --- | --- | --- | --- | --- | --- | --- | --- |
| CTL Healthy | 4447.26 ± 1434.71 | 72.38 ± 7.52 | 25.13 ± 7.16 | 0.00 | 0.00 | 1.15 ± 0.40 | 3325.29 ± 1314.43 | 1117.58 ± 284.11 | 0.00 | 0.00 | 51.09 ± 7.39 | 712.32 ± 91.99 | 6.77 ± 0.27 |
| Vehicle | 8463.67 ± 2208.71^****^ | 75.33 ± 3.26 | 22.49 ± 3.71 | 0.00 | 0.00 | 1.64 ± 0.75 | 6375.65 ± 1765.81^****^ | 1903.08 ± 526.89 | 0.00 | 0.00 | 139.12 ± 105.37 | 762.18 ± 59.88 | 6.42 |
| 4bt  (50 mg/Kg) | 6743.08 ± 1495.86^****^ | 67.94 ± 5.55 | 27.54 ± 6.62 | 0 ± 1.2 | 0.00 | 2.46 ± 1.33 | 4581.29 ± 1348.61^**^ | 1857.04 ± 257.84 | 0 ± 55.2 | 0.00 | 165.85 ± 129.48 | 651.72 ± 43.31 | 6.32 ± 0.10 |
| 4bc  (50 mg/Kg) | 6151.22 ± 1241.61^****^ | 64.44 ± 11.06 | 30.17 ± 10.71 | 0.00 | 0.00 | 2.30 ± 1.2 | 3963.88 ± 999.15 | 1855.79 ± 729.04 | 0.00 | 0.00 | 141.32 ± 105.13 | 703.92 ± 65.28 | 6.42 ± 0.1 |
| 4bt  (80 mg/kg) | 7571.58 ± 1981.58^****^ | 79.31 ± 1.70 | 18.55 ± 2.05 | 0 ± 0.47 | 0.00 | 1.44 ± 0.94 | 6005.40 ± 1684.47^****^ | 1404.59 ± 199.83 | 0 ± 29.7 | 0.00 | 109.20 ± 119.74 | 535.30 ± 207.08 | 6.83 ± 0.19 |
| 4bc  (80 mg/kg) | 4284.6 ± 1248.36 | 75.20 ± 5.46 | 23.25 ± 5.27 | 0.00 | 0.00 | 0 ± 0.40 | 3221.89 ± 1056.38 | 996.13 ± 258.89 | 0.00 | 0.00 | 0 ± 21.87 | 840.85 ± 102.58 | 6.56 ± 0.05 |
| 4bt+4bc  (25 mg/kg) | 5708.7 ± 1431.64^**^ | 68.80 ± 5.22 | 26.41 ± 4.75 | 0 ± 1.20 0 | 0 ± 0.4 | 1.89 ± 4.32 | 3927.62 ± 1224.18 | 1507.47 ± 339.32 | 0 ± 63.60 | 0 ± 21.20 | 107.79 ± 225.956 | 309.68 ± 313.72 | 6.37 ± 0.13 |
| Paclitaxel (20 mg/kg) | 2139.39 ± 684.75^****^ | 78.63 ± 2.36 | 12.07 ± 5.10 | 0.00 | 2 ± 1.25 | 4.79 ± 3.74 | 1682.24 ± 602.57^****^ | 258.30 ± 103.76 | 0.00 | 42.79 ± 22.69 | 102.51 ± 65.75 | 1070.01 ± 37.28 | 7.07 ± 0.12 |
| AC-T | 4236.11 ± 627.38 | 74.48 ± 4.13 | 24.12 ± 3.72 | 0.00 | 0 ± 0.4 | 0 ± 0.4 | 3155.28 ± 562.81 | 1021.78 ± 147.92 | 0.00 | 0 ± 16 | 0 ± 18.55 | 1066.24 ± 71.37 | 6.74 ± 0.05 |
| AC-4bt | 3609.98 ±  897.55 | 62.51 ± 10.84 | 32.48 ± 9.90 | 0 ± 0.4 | 0.00 | 0 ± 3.32 | 2256.45 ± 788.73^*^ | 1172.42 ± 353.10 | 0 ± 19.60 | 0.00 | 0 ± 167.74 | 830.47 ± 296.57 | 6.48 ± 0.10 |

WBC: white blood cells; MPV: mean platelet volume. Data represent mean ± SEM. ^*^*p* < 0.05, ^**^*p* < 0.01, ^***^*p* < 0.001, ^****^*p* < 0.0001 as compared to control group (CTL Healthy).
